# 2-(4-Chloro­phen­yl)imidazo[1,2-*a*]pyridine-3-carbaldehyde

**DOI:** 10.1107/S1600536809048302

**Published:** 2009-11-25

**Authors:** Yun-Hui Li, Wen-Yu Liu, Yang Gao, Yu-Peng Wang

**Affiliations:** a School of Chemistry and Environmental Engineering, Changchun University of Science and Technology, Changchun 130022, People’s Republic of China; b Jilin College of Transportation Vocation and Technology, Changchun 130012, People’s Republic of China

## Abstract

The asymmetric unit of the title compound, C_14_H_9_ClN_2_O, contains two mol­ecules with dihedral angles of 33.52 (11) and 34.58 (11)° between their benzene rings and imidazole ring systems. In the crystal, C—H⋯N and C—H⋯O inter­actions are observed. The crystal examined was found to be a racemic twin.

## Related literature

For the synthesis, see: Burkholder *et al.* (2001 ▶).
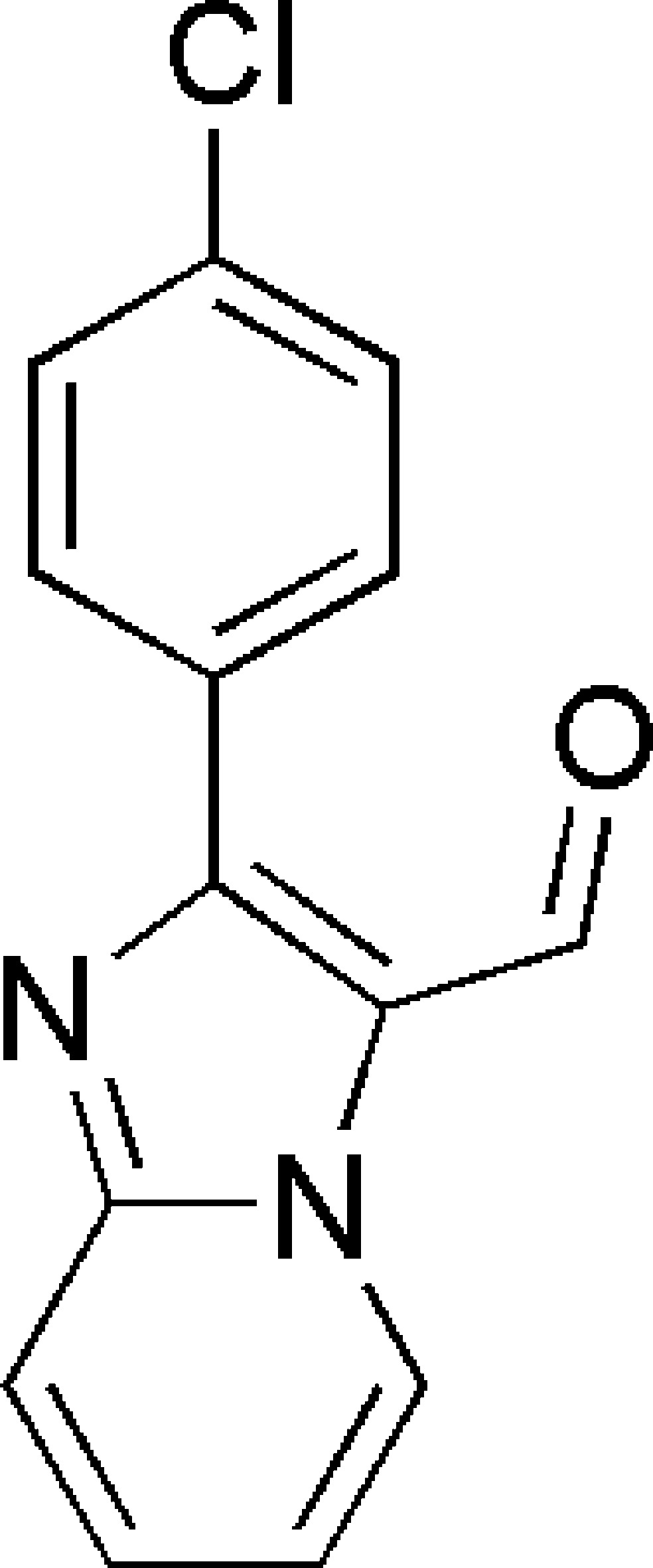


## Experimental

### 

#### Crystal data

C_14_H_9_ClN_2_O
*M* *_r_* = 256.68
Orthorhombic, 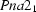

*a* = 21.3367 (13) Å
*b* = 7.2391 (4) Å
*c* = 15.1748 (10) Å
*V* = 2343.9 (2) Å^3^
*Z* = 8
Mo *K*α radiation
μ = 0.31 mm^−1^
*T* = 293 K
0.35 × 0.29 × 0.28 mm

#### Data collection

Bruker SMART APEX CCD diffractometer
Absorption correction: multi-scan (*SADABS*; Bruker, 2002 ▶) *T*_min_ = 0.870, *T*_max_ = 0.894
12428 measured reflections
4198 independent reflections
3564 reflections with *I* > 2σ(*I*)
*R*_int_ = 0.026

#### Refinement

*R*[*F* ^2^ > 2σ(*F* ^2^)] = 0.036
*wR*(*F* ^2^) = 0.089
*S* = 1.03
4198 reflections
325 parameters
1 restraint
H-atom parameters constrained
Δρ_max_ = 0.22 e Å^−3^
Δρ_min_ = −0.18 e Å^−3^
Absolute structure: Flack (1983 ▶), 1800 Friedel pairs
Flack parameter: 0.40 (5)

### 

Data collection: *SMART* (Bruker, 2002 ▶); cell refinement: *SAINT* (Bruker, 2002 ▶); data reduction: *SAINT*; program(s) used to solve structure: *SHELXS97* (Sheldrick, 2008 ▶); program(s) used to refine structure: *SHELXL97* (Sheldrick, 2008 ▶); molecular graphics: *SHELXTL* (Sheldrick, 2008 ▶); software used to prepare material for publication: *SHELXTL*.

## Supplementary Material

Crystal structure: contains datablocks global, I. DOI: 10.1107/S1600536809048302/hb5204sup1.cif

Structure factors: contains datablocks I. DOI: 10.1107/S1600536809048302/hb5204Isup2.hkl

Additional supplementary materials:  crystallographic information; 3D view; checkCIF report

## Figures and Tables

**Table 1:** Hydrogen-bond geometry (Å, °)

*D*—H⋯*A*	*D*—H	H⋯*A*	*D*⋯*A*	*D*—H⋯*A*
C11—H11⋯O2^i^	0.93	2.54	3.442 (3)	163
C12—H12⋯N4^ii^	0.93	2.59	3.518 (4)	172

Symmetry codes: (i) 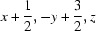; (ii) 
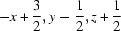
.

**Fig. 1.**
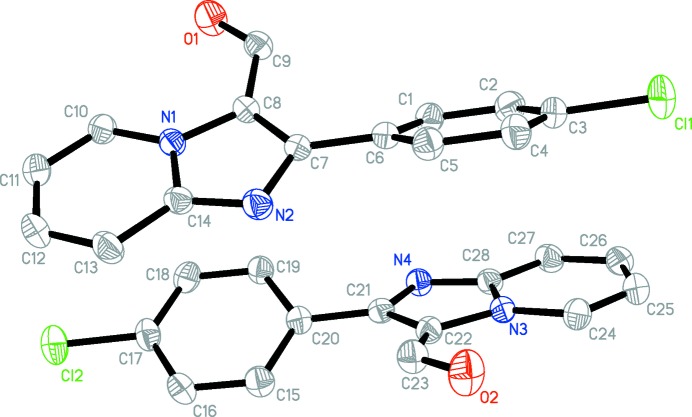
Molecular structure of (I) with displacement ellipsoids drawn at the 30% probability level for non-H atoms.

## supplementary crystallographic information

### Experimental

To a solution of 2.0 mmol 2-(4-chlorophenyl)H-imidazo[1,2-a]pyridine (Burkholder *et al.*, 2001) in DMF (10 ml) was added phosphoryl trichloride (2.2 mmol) in one portion at room temperature under stirring. The mixture was heated to 353 K and stirred for 5.0 h. After the intermediate was consumed (monitored by TLC), the reaction mixture was extracted, filtered and concentrated in vacuo. The pure product was obtained through silica gel chromatography, and yellow blocks of (I) were obtained by slow evaporation of an ethyl acetate/petroleum ether (1:1) solution at room temperature.

### Refinement

All H atoms were generated geometrically and refined as riding atoms with C—H = 0.93Å and *U*_iso_(H) = 1.2 *U*_eq_(C).

### Crystal data

**Table:**

C_14_H_9_ClN_2_O	*F*(000) = 1056
*M**_r_* = 256.68	*D*_x_ = 1.455 Mg m^−^^3^
Orthorhombic, *P**n**a*2_1_	Mo *K*α radiation, λ = 0.71073 Å
Hall symbol: P 2c -2n	Cell parameters from 4265 reflections
*a* = 21.3367 (13) Å	θ = 1.9–26.0°
*b* = 7.2391 (4) Å	µ = 0.31 mm^−^^1^
*c* = 15.1748 (10) Å	*T* = 293 K
*V* = 2343.9 (2) Å^3^	Block, yellow
*Z* = 8	0.35 × 0.29 × 0.28 mm

### Data collection

**Table:**

Bruker APEX CCD diffractometer	4198 independent reflections
Radiation source: fine-focus sealed tube	3564 reflections with *I* > 2σ(*I*)
graphite	*R*_int_ = 0.026
ω scans	θ_max_ = 26.0°, θ_min_ = 1.9°
Absorption correction: multi-scan (*SADABS*; Bruker, 2002)	*h* = −26→26
*T*_min_ = 0.870, *T*_max_ = 0.894	*k* = −8→8
12428 measured reflections	*l* = −15→18

### Refinement

**Table:**

Refinement on *F*^2^	Secondary atom site location: difference Fourier map
Least-squares matrix: full	Hydrogen site location: inferred from neighbouring sites
*R*[*F*^2^ > 2σ(*F*^2^)] = 0.036	H-atom parameters constrained
*wR*(*F*^2^) = 0.089	*w* = 1/[σ^2^(*F*_o_^2^) + (0.0461*P*)^2^ + 0.2155*P*] where *P* = (*F*_o_^2^ + 2*F*_c_^2^)/3
*S* = 1.03	(Δ/σ)_max_ < 0.001
4198 reflections	Δρ_max_ = 0.22 e Å^−^^3^
325 parameters	Δρ_min_ = −0.18 e Å^−^^3^
1 restraint	Absolute structure: Flack (1983), 1800 Friedel pairs
Primary atom site location: structure-invariant direct methods	Flack parameter: 0.40 (5)

### Special details

**Table:**

Geometry. All esds (except the esd in the dihedral angle between two l.s. planes) are estimated using the full covariance matrix. The cell esds are taken into account individually in the estimation of esds in distances, angles and torsion angles; correlations between esds in cell parameters are only used when they are defined by crystal symmetry. An approximate (isotropic) treatment of cell esds is used for estimating esds involving l.s. planes.
Refinement. Refinement of F^2^ against ALL reflections. The weighted R-factor wR and goodness of fit S are based on F^2^, conventional R-factors R are based on F, with F set to zero for negative F^2^. The threshold expression of F^2^ > 2sigma(F^2^) is used only for calculating R-factors(gt) etc. and is not relevant to the choice of reflections for refinement. R-factors based on F^2^ are statistically about twice as large as those based on F, and R- factors based on ALL data will be even larger.

### Fractional atomic coordinates and isotropic or equivalent isotropic displacement parameters (Å^2^)

**Table:**

	*x*	*y*	*z*	*U*_iso_*/*U*_eq_	
Cl1	0.38891 (3)	0.69710 (12)	0.18830 (6)	0.0741 (3)	
Cl2	0.86516 (3)	1.12252 (11)	0.50704 (6)	0.0673 (2)	
O2	0.48266 (8)	1.0458 (3)	0.48167 (13)	0.0652 (6)	
O1	0.76678 (8)	0.5045 (3)	0.22142 (13)	0.0620 (5)	
N1	0.73924 (8)	0.6319 (3)	0.39935 (13)	0.0373 (4)	
N2	0.64367 (8)	0.7006 (3)	0.45277 (14)	0.0434 (5)	
N4	0.60866 (9)	1.2130 (3)	0.24891 (14)	0.0403 (5)	
C6	0.57814 (10)	0.6745 (3)	0.32064 (17)	0.0387 (6)	
N3	0.51184 (8)	1.1689 (3)	0.30384 (13)	0.0368 (4)	
C7	0.63898 (10)	0.6637 (3)	0.36576 (16)	0.0345 (5)	
C21	0.61298 (10)	1.1699 (3)	0.33495 (17)	0.0370 (5)	
C11	0.83204 (12)	0.6244 (4)	0.48197 (19)	0.0507 (7)	
H11	0.8750	0.6038	0.4857	0.061*	
C17	0.79144 (11)	1.1336 (4)	0.45777 (19)	0.0467 (6)	
C14	0.70483 (10)	0.6813 (3)	0.47280 (16)	0.0400 (6)	
C20	0.67494 (10)	1.1586 (3)	0.37871 (17)	0.0376 (6)	
C8	0.69729 (10)	0.6195 (3)	0.32942 (16)	0.0360 (5)	
C28	0.54724 (10)	1.2129 (3)	0.23030 (16)	0.0384 (6)	
C18	0.78521 (11)	1.0860 (4)	0.37049 (19)	0.0489 (6)	
H18	0.8198	1.0467	0.3382	0.059*	
C22	0.55419 (10)	1.1401 (3)	0.37297 (16)	0.0380 (5)	
C19	0.72678 (10)	1.0975 (3)	0.33157 (17)	0.0423 (6)	
H19	0.7221	1.0638	0.2728	0.051*	
C2	0.51455 (11)	0.7437 (4)	0.1929 (2)	0.0505 (6)	
H2	0.5110	0.7846	0.1350	0.061*	
C9	0.71397 (11)	0.5482 (3)	0.24447 (17)	0.0434 (6)	
H9	0.6819	0.5340	0.2035	0.052*	
C5	0.52395 (11)	0.6241 (3)	0.36600 (18)	0.0448 (6)	
H5	0.5268	0.5846	0.4242	0.054*	
C24	0.44781 (11)	1.1572 (3)	0.30118 (19)	0.0462 (6)	
H24	0.4251	1.1274	0.3515	0.055*	
C27	0.51591 (12)	1.2473 (4)	0.15048 (19)	0.0492 (6)	
H27	0.5384	1.2786	0.1001	0.059*	
C13	0.73559 (11)	0.7049 (4)	0.55341 (19)	0.0507 (7)	
H13	0.7133	0.7401	0.6033	0.061*	
C10	0.80304 (10)	0.6045 (3)	0.40399 (19)	0.0446 (6)	
H10	0.8257	0.5727	0.3539	0.054*	
C26	0.45240 (12)	1.2345 (4)	0.14768 (19)	0.0526 (7)	
H26	0.4314	1.2554	0.0949	0.063*	
C1	0.57237 (11)	0.7353 (4)	0.23404 (18)	0.0439 (6)	
H1	0.6080	0.7709	0.2032	0.053*	
C16	0.74146 (11)	1.1959 (4)	0.5060 (2)	0.0497 (6)	
H16	0.7466	1.2286	0.5649	0.060*	
C4	0.46599 (12)	0.6322 (4)	0.32548 (19)	0.0497 (6)	
H4	0.4300	0.5988	0.3561	0.060*	
C23	0.53638 (11)	1.0703 (3)	0.45701 (17)	0.0463 (6)	
H23	0.5683	1.0410	0.4963	0.056*	
C15	0.68309 (11)	1.2097 (4)	0.46614 (18)	0.0468 (6)	
H15	0.6491	1.2536	0.4983	0.056*	
C12	0.79878 (12)	0.6758 (4)	0.5579 (2)	0.0513 (7)	
H12	0.8197	0.6899	0.6112	0.062*	
C25	0.41833 (12)	1.1899 (4)	0.22393 (19)	0.0514 (7)	
H25	0.3749	1.1827	0.2212	0.062*	
C3	0.46229 (12)	0.6899 (4)	0.23968 (19)	0.0485 (7)	

### Atomic displacement parameters (Å^2^)

**Table:**

	*U*^11^	*U*^22^	*U*^33^	*U*^12^	*U*^13^	*U*^23^
Cl1	0.0499 (4)	0.0888 (6)	0.0835 (6)	0.0081 (4)	−0.0283 (4)	−0.0004 (5)
Cl2	0.0459 (3)	0.0834 (5)	0.0725 (5)	−0.0041 (3)	−0.0179 (3)	0.0060 (4)
O2	0.0454 (10)	0.0979 (15)	0.0524 (12)	−0.0022 (10)	0.0123 (9)	0.0151 (11)
O1	0.0459 (10)	0.0937 (16)	0.0462 (12)	0.0041 (10)	0.0086 (9)	−0.0156 (10)
N1	0.0354 (10)	0.0400 (11)	0.0365 (12)	−0.0013 (8)	−0.0023 (9)	−0.0030 (8)
N2	0.0392 (10)	0.0512 (13)	0.0399 (14)	0.0004 (9)	0.0005 (10)	−0.0062 (10)
N4	0.0380 (10)	0.0492 (12)	0.0337 (12)	−0.0002 (9)	0.0021 (9)	−0.0003 (9)
C6	0.0399 (13)	0.0367 (12)	0.0393 (15)	0.0020 (10)	−0.0026 (11)	−0.0046 (11)
N3	0.0343 (10)	0.0400 (10)	0.0361 (12)	0.0006 (8)	−0.0024 (9)	−0.0024 (8)
C7	0.0354 (11)	0.0351 (12)	0.0331 (13)	0.0008 (9)	−0.0014 (10)	−0.0026 (10)
C21	0.0393 (12)	0.0365 (12)	0.0354 (14)	0.0017 (10)	0.0030 (11)	−0.0013 (10)
C11	0.0389 (12)	0.0547 (16)	0.0585 (19)	0.0020 (11)	−0.0094 (13)	−0.0043 (13)
C17	0.0409 (13)	0.0476 (15)	0.0517 (18)	−0.0066 (11)	−0.0098 (13)	0.0087 (12)
C14	0.0388 (12)	0.0448 (14)	0.0364 (15)	0.0006 (11)	0.0010 (11)	−0.0068 (10)
C20	0.0357 (12)	0.0385 (13)	0.0384 (15)	−0.0040 (9)	−0.0001 (10)	0.0044 (11)
C8	0.0366 (12)	0.0379 (12)	0.0337 (13)	−0.0032 (9)	−0.0010 (10)	0.0000 (10)
C28	0.0377 (12)	0.0444 (14)	0.0333 (14)	−0.0012 (10)	0.0025 (10)	−0.0032 (10)
C18	0.0387 (13)	0.0547 (17)	0.0533 (18)	0.0038 (11)	0.0035 (12)	0.0056 (13)
C22	0.0352 (12)	0.0413 (13)	0.0375 (14)	0.0011 (9)	0.0005 (11)	−0.0015 (10)
C19	0.0411 (13)	0.0500 (14)	0.0359 (14)	0.0018 (10)	0.0011 (11)	0.0014 (11)
C2	0.0561 (15)	0.0541 (17)	0.0411 (16)	0.0085 (12)	−0.0045 (14)	0.0009 (13)
C9	0.0438 (13)	0.0479 (15)	0.0385 (14)	−0.0032 (11)	0.0001 (11)	−0.0027 (11)
C5	0.0418 (13)	0.0505 (15)	0.0421 (15)	0.0007 (10)	0.0016 (11)	−0.0004 (12)
C24	0.0359 (12)	0.0513 (15)	0.0513 (17)	−0.0028 (11)	0.0038 (12)	−0.0016 (12)
C27	0.0536 (15)	0.0571 (16)	0.0368 (16)	−0.0002 (12)	−0.0031 (13)	−0.0029 (12)
C13	0.0513 (15)	0.0651 (17)	0.0357 (16)	0.0014 (13)	−0.0052 (13)	−0.0103 (13)
C10	0.0341 (12)	0.0499 (15)	0.0497 (16)	0.0014 (11)	0.0010 (11)	−0.0045 (12)
C26	0.0545 (15)	0.0571 (16)	0.0463 (18)	0.0074 (13)	−0.0121 (14)	−0.0076 (13)
C1	0.0408 (13)	0.0475 (15)	0.0435 (17)	0.0017 (11)	0.0008 (11)	0.0011 (11)
C16	0.0509 (14)	0.0592 (16)	0.0389 (16)	−0.0058 (12)	−0.0078 (13)	−0.0015 (13)
C4	0.0378 (13)	0.0559 (16)	0.0555 (18)	−0.0022 (11)	−0.0025 (12)	−0.0025 (13)
C23	0.0425 (13)	0.0539 (15)	0.0424 (15)	−0.0009 (11)	0.0028 (12)	0.0038 (12)
C15	0.0449 (13)	0.0561 (17)	0.0395 (17)	−0.0011 (11)	0.0036 (12)	0.0015 (12)
C12	0.0503 (15)	0.0621 (17)	0.0414 (16)	−0.0041 (13)	−0.0133 (13)	−0.0032 (13)
C25	0.0402 (13)	0.0579 (17)	0.0560 (18)	0.0024 (12)	−0.0091 (13)	−0.0064 (13)
C3	0.0421 (13)	0.0475 (15)	0.0560 (18)	0.0069 (11)	−0.0129 (13)	−0.0065 (13)

### Geometric parameters (Å, °)

**Table:**

Cl1—C3	1.750 (3)	C28—C27	1.406 (4)
Cl2—C17	1.743 (2)	C18—C19	1.382 (3)
O2—C23	1.219 (3)	C18—H18	0.9300
O1—C9	1.221 (3)	C22—C23	1.424 (3)
N1—C10	1.378 (3)	C19—H19	0.9300
N1—C14	1.382 (3)	C2—C3	1.378 (4)
N1—C8	1.391 (3)	C2—C1	1.384 (3)
N2—C14	1.347 (3)	C2—H2	0.9300
N2—C7	1.351 (3)	C9—H9	0.9300
N4—C28	1.341 (3)	C5—C4	1.382 (3)
N4—C21	1.346 (3)	C5—H5	0.9300
C6—C1	1.391 (4)	C24—C25	1.351 (4)
C6—C5	1.394 (3)	C24—H24	0.9300
C6—C7	1.470 (3)	C27—C26	1.359 (3)
N3—C24	1.369 (3)	C27—H27	0.9300
N3—C28	1.385 (3)	C13—C12	1.366 (3)
N3—C22	1.400 (3)	C13—H13	0.9300
C7—C8	1.398 (3)	C10—H10	0.9300
C21—C22	1.397 (3)	C26—C25	1.404 (4)
C21—C20	1.482 (3)	C26—H26	0.9300
C11—C10	1.343 (4)	C1—H1	0.9300
C11—C12	1.403 (4)	C16—C15	1.389 (3)
C11—H11	0.9300	C16—H16	0.9300
C17—C16	1.370 (4)	C4—C3	1.370 (4)
C17—C18	1.375 (4)	C4—H4	0.9300
C14—C13	1.399 (4)	C23—H23	0.9300
C20—C15	1.388 (4)	C15—H15	0.9300
C20—C19	1.390 (3)	C12—H12	0.9300
C8—C9	1.434 (3)	C25—H25	0.9300
			
C10—N1—C14	121.4 (2)	C1—C2—H2	120.8
C10—N1—C8	131.7 (2)	O1—C9—C8	125.4 (2)
C14—N1—C8	106.87 (18)	O1—C9—H9	117.3
C14—N2—C7	105.8 (2)	C8—C9—H9	117.3
C28—N4—C21	105.7 (2)	C4—C5—C6	120.8 (3)
C1—C6—C5	118.4 (2)	C4—C5—H5	119.6
C1—C6—C7	122.3 (2)	C6—C5—H5	119.6
C5—C6—C7	119.2 (2)	C25—C24—N3	118.6 (2)
C24—N3—C28	122.3 (2)	C25—C24—H24	120.7
C24—N3—C22	131.1 (2)	N3—C24—H24	120.7
C28—N3—C22	106.61 (18)	C26—C27—C28	119.3 (3)
N2—C7—C8	111.4 (2)	C26—C27—H27	120.4
N2—C7—C6	120.7 (2)	C28—C27—H27	120.4
C8—C7—C6	127.9 (2)	C12—C13—C14	119.2 (2)
N4—C21—C22	112.0 (2)	C12—C13—H13	120.4
N4—C21—C20	120.6 (2)	C14—C13—H13	120.4
C22—C21—C20	127.4 (2)	C11—C10—N1	119.0 (2)
C10—C11—C12	121.3 (2)	C11—C10—H10	120.5
C10—C11—H11	119.4	N1—C10—H10	120.5
C12—C11—H11	119.4	C27—C26—C25	120.4 (3)
C16—C17—C18	121.5 (2)	C27—C26—H26	119.8
C16—C17—Cl2	119.2 (2)	C25—C26—H26	119.8
C18—C17—Cl2	119.3 (2)	C2—C1—C6	121.2 (2)
N2—C14—N1	111.1 (2)	C2—C1—H1	119.4
N2—C14—C13	129.7 (2)	C6—C1—H1	119.4
N1—C14—C13	119.2 (2)	C17—C16—C15	119.2 (3)
C15—C20—C19	118.5 (2)	C17—C16—H16	120.4
C15—C20—C21	121.7 (2)	C15—C16—H16	120.4
C19—C20—C21	119.8 (2)	C3—C4—C5	119.2 (3)
N1—C8—C7	104.9 (2)	C3—C4—H4	120.4
N1—C8—C9	123.3 (2)	C5—C4—H4	120.4
C7—C8—C9	131.1 (2)	O2—C23—C22	125.3 (2)
N4—C28—N3	111.3 (2)	O2—C23—H23	117.3
N4—C28—C27	130.3 (2)	C22—C23—H23	117.3
N3—C28—C27	118.4 (2)	C20—C15—C16	120.7 (2)
C17—C18—C19	118.9 (2)	C20—C15—H15	119.7
C17—C18—H18	120.5	C16—C15—H15	119.7
C19—C18—H18	120.5	C13—C12—C11	120.0 (3)
C21—C22—N3	104.3 (2)	C13—C12—H12	120.0
C21—C22—C23	131.6 (2)	C11—C12—H12	120.0
N3—C22—C23	123.5 (2)	C24—C25—C26	121.0 (2)
C18—C19—C20	121.1 (2)	C24—C25—H25	119.5
C18—C19—H19	119.4	C26—C25—H25	119.5
C20—C19—H19	119.4	C4—C3—C2	122.0 (2)
C3—C2—C1	118.5 (3)	C4—C3—Cl1	119.0 (2)
C3—C2—H2	120.8	C2—C3—Cl1	119.1 (2)

### Hydrogen-bond geometry (Å, °)

**Table:**

*D*—H···*A*	*D*—H	H···*A*	*D*···*A*	*D*—H···*A*
C11—H11···O2^i^	0.93	2.54	3.442 (3)	163
C12—H12···N4^ii^	0.93	2.59	3.518 (4)	172

Symmetry codes: (i) *x*+1/2, −*y*+3/2, *z*; (ii) −*x*+3/2, *y*−1/2, *z*+1/2.
